# Two species of Naididae (Annelida, Clitellata) from southern Tibet, China

**DOI:** 10.3897/zookeys.444.8285

**Published:** 2014-10-07

**Authors:** Yu Peng, Hongzhu Wang, Yongde Cui

**Affiliations:** 1 State Key Laboratory of Freshwater Ecology and Biotechnology, Institute of Hydrobiology, Chinese Academy of Sciences, Wuhan 430072, China; 2 University of Chinese Academy of Sciences, Beijing 100049, China

**Keywords:** *Nais badia*, *Tubifex montanus*, Naididae, new species, new record species, taxonomy, southern Tibet

## Abstract

One new species of Naidinae (Oligochaeta, Naididae), *Nais badia* **sp. n.** and one new record species from China, *Tubifex montanus* Kowalewski, 1919 (Tubificinae) are found in southern Tibet. The new species is distinguished from congeners by its large area of reddish brown pigment in the anterior segments I–VIII, serrate hairs, pectinate needles with 1–2 intermediate teeth, ventral chaetae partly with 1–2 fine intermediate teeth and wave-like movements. The new material of the species *Tubifex montanus* differs slightly from the previous descriptions by its vas deferens entering atrium subapically, wide ental end of penial sheath and smooth hair chaetae.

## Introduction

The Tibetan Plateau is one of the biodiversity hotspots around the globe for its unique natural environment (Li and Fang 1999), which accounts for the rich occurrence of endemic species of various taxa in this region, such as *Triplophysa cakaensis* (Cobitidae) (Cao and Zhu 1988), *Schizothorax waltoni* (Cyprinidae) (Chen and Chen 2010), and *Alona aliensis* (Chydoridae) (Chiang et al. 1983). What about oligochaetes? In the 20th century, there were only seven species of oligochaetes recorded in Tibet (Stephenson 1909; Černosvitov 1942; Liang 1963, 1979; Liang et al. 1998). Recently, He recorded 26 species in Tibet (He 2011; He et al. 2012), though focusing mainly on large rivers (Yarlung Zangbo River) and brackish lakes (Lake Nam Co and Lake Yamzho Yumco). Some freshwater wetlands among mountains in Tibet had been neglected, which we thought may be the ideal habitats for aquatic oligochaetes. In this paper, we describe one new species, *Nais badia* sp. n., and one new record of *Tubifex montanus* Kowalewski, 1919 found in a freshwater wetland of Cuomujiri Mountain, southern Tibet.

## Materials and methods

The sampling site was in a wetland of Cuomujiri Mountain, southern Tibet of China. (29°47'46"N, 94°24'53"E), ca 3,990 m above sea level. The substrate type was silt, and hydrophytes were abundant. Water depth was 10–20 cm, water temperature 11.5 °C, pH 6.23, dissolved oxygen 9.14 mg/L, and conductivity 19.3 µs/cm.

The samples were collected with a D-frame dip net, and cleaned through a 250 µm sieve. Large worms were sorted in a white porcelain dish manually and small individuals were sorted with a dissecting microscope. Specimens were all preserved in 10% formalin. Some specimens were investigated with a Scanning Election Microscopy (SEM) to reveal more details of the chaetae. Some were stained with borax carmine, dehydrated in a series of alcohol, cleared in xylene and mounted whole in Canada balsam for careful observation. Parameters of external morphology were established under glycerine mounts. Other parameters were studied on permanent mounts. Drawings were made with a camera lucida. All microscopic observations, including live observations, were documented photographically. The types and other specimens were all deposited in the Institute of Hydrobiology (IHB), Chinese Academy of Sciences (CAS), Wuhan, China.

### Abbreviations in the figures

Roman numbers = segment number, SEM = Scanning Electron Microscopy, at = atrium, mp = male pore, pe = penis, pr = prostate gland, ps = penial sheath, sf = sperm funnel, sp = spermatheca, spp = spermathecal pore, vd = vas deferens.

## Taxonomy

### Family Naididae

#### Subfamily Naidinae

##### Nais badia sp. n.

Taxon classificationAnimaliaHaplotaxidaNaididae

http://zoobank.org/B20893C6-27F2-4BF1-8A63-FE6EDA75BCF6

**Figure 1.**
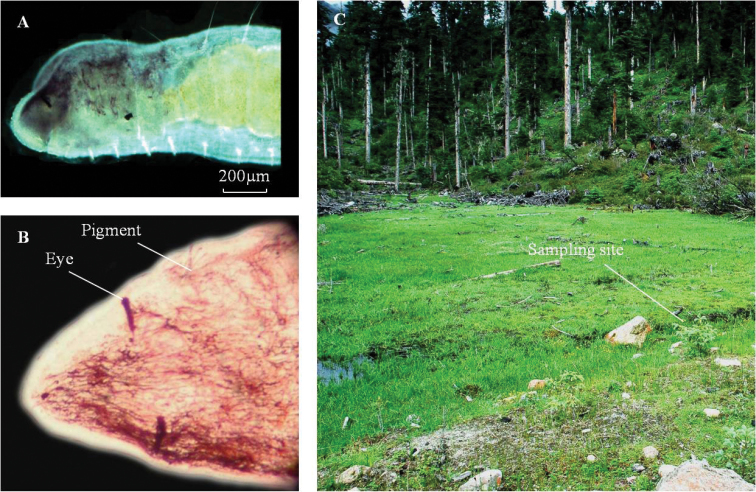
*Nais badia* sp. n. **A** anterior reddish brown pigment in segments I-VIII **B** dorsal view of the head, live worm **C** habitat.

**Figure 2.**
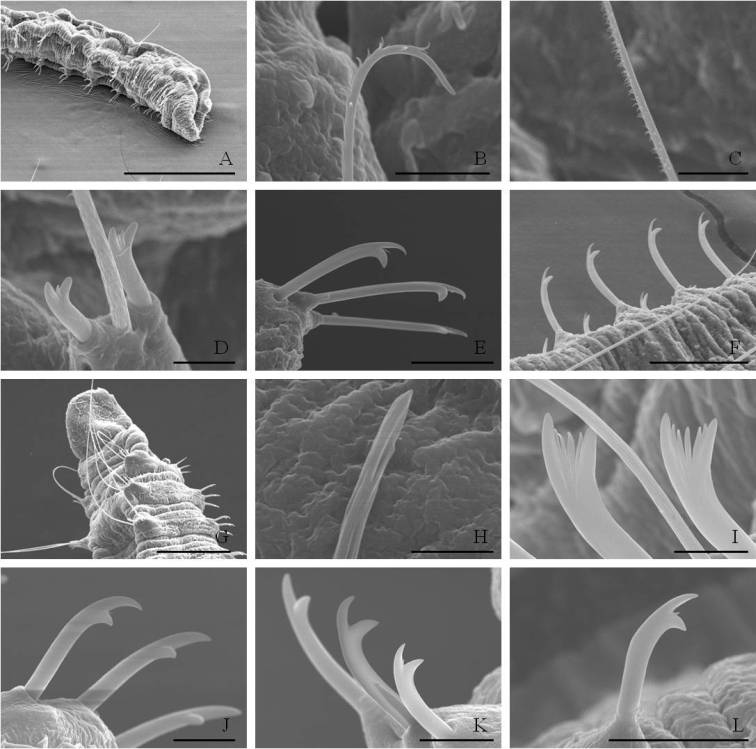
SEM micrographs **A–F** *Nais badia* sp. n. **A** lateral view of anterior body **B–C** hairs in VIII and VI **D** needles in VI **E–F** ventral chaetae in XIII and posterior. **G–L** *Tubifex montanus* **G** lateral view of the head **H** hairs in X **I** needles in II **J–L** ventral chaetae in IV, XIII and posterior. Scale bar: **A** 300 µm, **B, D** and **I** 5 µm, **C, J** and **K** 10 µm, **E** and **L** 20 µm, **F** 40 µm, **G** 100 µm, **H** 2 µm.

**Figure 3.**
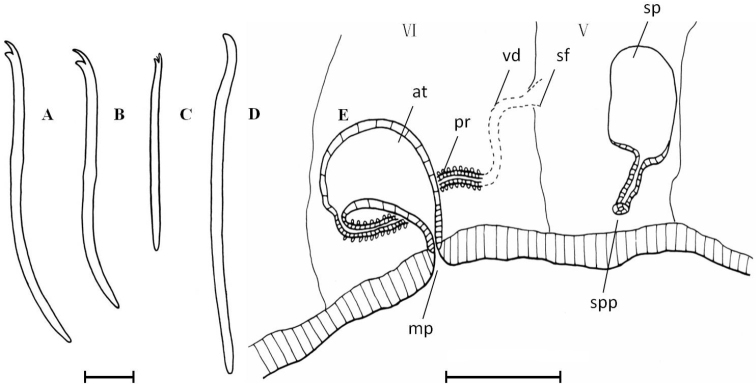
*Nais badia* sp. n. **A–B** ventral chaetae in V and X **C** dorsal bifid in X **D** penial chaeta in VI **E** lateral view of male duct and spermatheca in segments V–VI. Scale bar: **A–D** 20 µm, **E** 120 µm.

Figs 1 , 2 , 3 ; Table 1

###### Holotype.

IHB XZ20130630a, whole-mounted specimen, immature.

###### Type locality.

Wetland in Cuomujiri Mountain (29°47'46"N, 94°24'53"E), southern Tibet of China. 30 June 2013, collected by H.Z. Wang, Y.D. Cui, Y.J. He and Y. Peng.

###### Paratypes.

IHB XZ20130630b-f, 2 whole-mounted specimens (mature), 2 whole-mounted specimens (immature), 1 specimen is used for scanning electron microscopy. 30 June 2013, collected from the type locality.

###### Other material.

40 specimens are preserved in 10% formalin. 30 June 2013, collected from the type locality.

###### Etymology.

The specific name “*badia*” is Latin for “badius”, and refers to this worm’s large area of reddish brown pigment in anterior segments I-VIII.

###### Description.

Length 4.2–9.1 mm (Holotype 7.2 mm), width at V 0.3–0.6 mm (Holotype 0.6 mm). Segments 24–54 (Holotype 52). Prostomium conical, eyes present, large area of reddish brown pigment in segments I-VIII (Fig. 1A–B). Clitellum inconspicuous. Coelomocytes present. Stomach dilatation sudden in VII–VIII. Wave-like movements.

Dorsal chaetae beginning in VI onwards (Fig. 2A). Hairs (0)1–3 per bundle, 137–325 µm long, all serrate (Fig. 2B–C). Needles (0)2–3 per bundle, 80–90 µm long, distal tooth slightly longer than the proximal one (VII 3.8 µm/3.2 µm), completely pectinate with 1–2 intermediate teeth (Fig. 2D), nodulus often inconspicuous, 1/3 from the distal end (Fig. 3C). Ventral chaetae in II-V 7–8 per bundle, the rest 2–6(7) per bundle, 105–128 µm long, distal tooth longer and thinner than the proximal one (7.5 µm/5 µm), about 50% of ventral chaetae with 1–2 fine intermediate teeth (Fig. 2E–F), nodulus median or slightly distal (Fig. 3A–B). Penial chaetae 4 on each side in VI, with a simple hook, 115–155 µm long, 4.2–6.3 µm thick (Fig. 3D).

Pharyngeal glands in II–III. Clitellum in V–VI. Male genitalia paired in V–VI (Fig. 3E). Vasa deferentias 260–273 μm long, with prostate gland cells covering only on their posterior part, join atria subapically (Fig. 3E, vd and pr). Atrial ampullae large and ovoid, 150–160 μm long, 70–90 μm wide, ducts short and narrow (Fig. 3E, at). Spermathecal ampullae globular, length 80–100 µm, width 75–90 µm, spermathecal ducts long and narrow, length 60–70 µm, width 15–16 µm (Fig. 3E, sp).

###### Distribution.

Known only from Cuomujiri Mountain, southern Tibet of China. High mountain, wetland, hydrophytes abundant (Fig. 1C).

###### Remarks.

The presence of eyes, dorsal chaetae beginning in VI consisting of hairs and double-pronged needles, pharynx in II-III, stomach beginning in VII, coelomocytes present, spermathecae with distinct ducts, male ducts paired in V–VI, vas deferens with prostate glands joining atrium subapically, atrium without prostate, penial chaetae present with a simple hook, indicate that this new species fits the definition of *Nais* Müller, 1773 (Sperber 1948; Brinkhurst and Jamieson 1971).

*Nais badia* sp. n. is distinguished from congeners for having a large area of reddish brown pigment in anterior segments I-VIII, hairs all serrate, needles pectinate with 1–2 intermediate teeth, ventral chaetae with (0)1–2 fine intermediate teeth (Only visible under SEM) and wave-like movements (Table 1). We are hence of the opinion that it can be described as new to science.

**Table 1.** Comparison of *Nais badia* sp. n. with allied species.

Species		*Nais badia* sp. n.	*Nais africana*	*Nais elinguis*	*Nais communis*	*Nais variabilis*	*Nais pardalis*	*Nais bretscheri*
Pigment		Reddish brown in I-VIII	-	Anterior end reddish brown	Brown in I-V	Pigment in I-V or absent	Brown anteriorly	Anterior end heavily pigmented
Stomach		Dilatation sudden	-	Dilatation gradual	Dilatation gradual	Dilatation sudden	Dilatation sudden, with elongated cells	Dilatation gradual
Swimming		Wave-like movements	-	Lateral movements	No swimming	Spiral movements	Spiral movements	Spiral movements
Penial chaetae		4	Present	4–5	2–3	2–3	3	2
Ventral chaetae	Number	3–8	3–4	2–5	2–6	2–7	1–5	1–7
	II-V & rest	Similar	Different	Similar	Similar	Different	Different	Different
Hairs		1–3, serrate	1–2	1–3	1–2	1–2	1–2	1–2
Needles	Number	2–3, pectinate	1–2, pectinate	1–3	1–2	1–2	1–2	1–2
	Shape	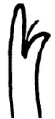	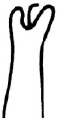	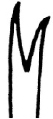	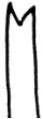	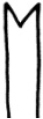	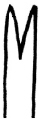	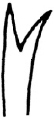
Spermathecae		Roundish, duct long and narrow	-	Large and elongated, duct long and narrow	Large, duct narrow	Ovoid, duct strong and dilated	Ovoid, duct well defined with a distal dwelling	Duct not dilated
Male ducts	Vasa deferentia	Surrounded by abundant gland cells only on their posterior part	-	Completely surrounded by abundant gland cells	Thick with prostate on their posterior part	Surrounded by strong gland cells on their posterior part	Surrounded by prostate gland cells in front of the atria	With prostate glands
	Atria	Pear-shaped, duct short and narrow	-	Globular, duct long and wide, well defined	Roundish, as long as duct	Pear-shaped, duct short and narrow or swollen	Pear-shaped, duct poorly defined and narrow	Globular
Distribution		Southern Tibet, China	Skoonspruit, Transvaal	Cosmopolitan	Cosmopolitan	Cosmopolitan	Cosmopolitan	Cosmopolitan
References		Present research	Brinkhurst 1966	Sperber 1948; Brinkhurst and Jamieson 1971; Semernoy 2004				

This new species is similar to *Nais africana* Brinkhurst, 1966 for having pectinate needles, but differs from it by its ventral chaetae in II-V differing from the rest, although the position of *Nais africana* in the genus is regarded as uncertain due to a lack of detailed examination of live worms and sectioned materials (Brinkhurst 1966). *Nais elinguis* resembles the new species on the needles and simple pointed penial chaetae, but its long and wide atrial duct, slow stomach dilatations and the vas deferens which is completely surrounded by abundant prostate gland cells are significantly different from the new species. With regard to the vas deferens completely surrounded by prostate gland cells on their posterior part, this new species is similar to *Nais communis*, *Nais variabilis* and *Nais pardalis*. However, some characteristic features of these species distinguish them from the new species. *Nais communis* eyes are generally absent, stomach dilatations are slow and the atrium is as long as the duct. *Nais variabilis*, *Nais pardalis* and *Nais bretscheri* all have the ventral chaetae in II-V that differ from the remaining segments. *Nais pardalis* the stomach has obvious elongated cells. *Nais bretscheri* the ventral chaetae have typical giant chaetae.

#### Subfamily Tubificinae

##### Tubifex montanus

Taxon classificationAnimaliaHaplotaxidaNaididae

Kowalewski, 1919

**Figure 4.**
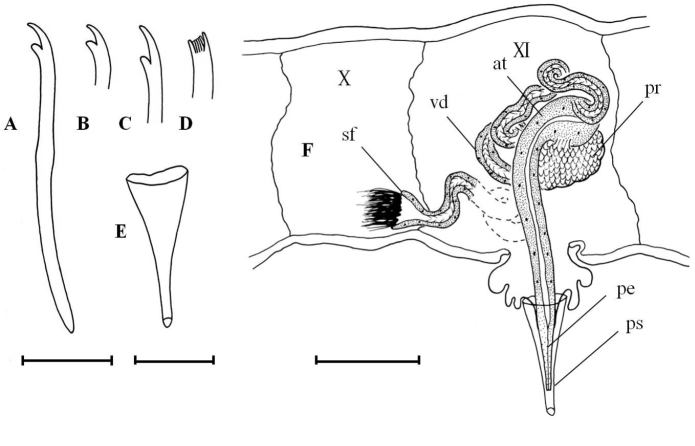
*Tubifex montanus* **A–C** ventral chaetae in II, XVIII and XXIX **D** dorsal chaetae in VI **E** penial sheath **F** lateral view of male duct in segments X–XI. Scale bar: **A–D** 20 µm, **E** 80 µm, **F** 160 µm.

Figs 2 , 4 ; Table 2

Tubifex montanus Kowalewski: Hrabě 1939, 1981; Brinkhurst and Jamieson 1971.

###### Examined material.

IHB XZ20130630g-i, 2 whole-mounted mature specimens although only 1 specimen has male ducts that can be observed and measured, 1 specimen is used for scanning electron microscopy. Wetland in Cuomujiri Mountain (29°47'46"N, 94°24'53"E) of southern Tibet, China. 30 June 2013, collected by H.Z. Wang, Y.D. Cui, Y.J. He and Y. Peng.

###### Description.

Length 10–12 mm, width at XI 0.4–0.7 mm. Segments 41–56. Prostomium obtuse. Clitellum inconspicuous. No coelomocytes.

Dorsal chaetae (0)1–3 hairs and 1–3 needles per bundle. Hairs smooth (Fig. 2H–I), 180–463 µm long. Needles almost palmate (3–11 teeth or more, Fig. 2I and Fig. 4D), 40–100 µm long, two short outer teeth nearly equal (7.5 µm/7.5 µm). Ventral chaetae 60–100 µm long with (0)1–2 fine intermediate teeth partly (Fig. 2J–L and Fig. 4A–C), anteriorly 3–4 per bundle with upper tooth slightly thinner than and nearly 2–3 times as long as the lower (7.5 µm/3 µm), in midbody 1–2 per bundle with two nearly equal teeth (5 µm/5 µm), posteriorly 1–2 per bundle with upper tooth nearly 2 to 3 times as long as the lower (5 µm/2 µm). Ventral chaetae in XI present but unmodified.

Pharyngeal glands in II–V. Chloragogen cells beginning in VI onwards. Male ducts paired in X-XI (Fig. 4F). Vas deferens 722 µm long or more, nearly 2.2 times longer than the atrium, uniform and forming numerous loops in XI, ciliated throughout and entering narrow atrium subapically (Fig. 4F, vd). Atrium pear-shaped, with quite long ejaculatory duct, and gradually becomes narrower toward the ectal end, 343 µm long (Fig. 4F, at). Large compact prostate gland empties into the atrial ampulla near the sperm duct outlet, 137 µm long, 83 µm wide (Fig. 4F, pr). Penis cylindrical, 132 µm long, surrounded by a cuticularized, funnel-like penial sheath (Fig. 4E), 172 µm long and 71 µm wide at the ental end. Spermatheca absent. Testes paired in X. Ovaries paired in XI.

###### Distribution.

Cuomojiri Mountain, southern Tibet of China. A wetland with abundant hydrophytes. Tatra Mountains, Europe (Hrabě 1939; Brinkhurst and Jamieson 1971).

###### Remarks.

According to the characteristics of a long vas deferens entering pear-shaped atrium subapically, large compact prostate gland with stalk-like attachments to atrium and penis with funnel-like penial sheath, the species fits the definition of *Tubifex* Lamarck, 1816 (Brinkhurst and Jamieson 1971).

The new material resembles *Tubifex montanus* Kowalewski, 1919 in absence of spermathecae, vas deferens nearly of a similar length with cilia throughout, pear-shaped atrium with quite long ejaculatory duct gradually becoming narrower toward the ectal end, large compact prostate gland empting into the atrial ampullae near the sperm duct outlet, cylindrical penis, surrounded by cuticularized and funnel-like penial sheath, and nearly the same type of dorsal ventral chaetae (Table 2).

**Table 2.** Comparison of *Tubifex montanus* from Tibet and Europe.

Regions		Tibet	Europe
Body length (mm)		10–12	8 to 12
Body width (mm)		0.4–0.7	0.7
Segment number		41–56	40 to 50
Chaetae		Hairs smooth (1–3), needles palmate (1–3), ventral chaetae serrate partly (1–4)	Hairs hispid (2–3), needles palmate (2–3), ventral chaetae serrate (3–5)
Male ducts	Vasa deferentia	Ciliated along entire length, all one width, entering narrow atrium subapically, nearly 2.2 times longer than the atrium.	Ciliated along entire length, all one width, entering narrow atrium apically, nearly 1.5 times longer than the atrium.
	Atria	Pear-shaped	Asymmetrical, pear-shaped
	Prostate glands	Large compact prostate with stalk-like attachments to atrial ampulla near sperm duct outlet	Large compact prostate empties into the atrial ampulla near sperm duct outlet
	Penial sheaths	Funnel-like, cuticular tube	Conical, somewhat bent cuticular tube
Drawing		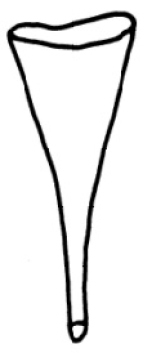 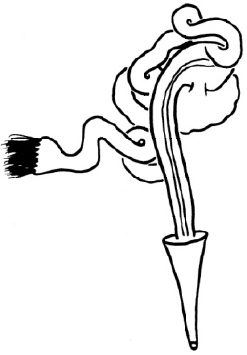	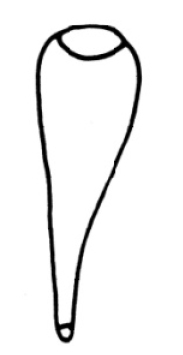 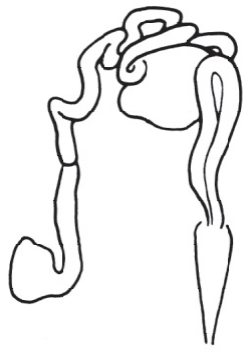
Spermathecae		Absent	Absent
Distribution		Cuomujiri Mountain, southern Tibet, China	Tatra Mountains, Europe
References		Present research	Hrabě 1939, 1981; Brinkhurst and Jamieson 1971

However, the new material differs slightly from the description by Hrabě (1939, 1981) by having the vas deferens entering the atrium subapically, straight penis sheath with ental end wider and smooth hair chaetae.

## Supplementary Material

XML Treatment for Nais badia

XML Treatment for Tubifex montanus
